# Biological treatment of pediatric sarcomas by combined virotherapy and NK cell therapy

**DOI:** 10.1186/s12885-019-6387-5

**Published:** 2019-12-03

**Authors:** Chihab Klose, Susanne Berchtold, Marina Schmidt, Julia Beil, Irina Smirnow, Sascha Venturelli, Markus Burkard, Rupert Handgretinger, Ulrich M. Lauer

**Affiliations:** 1 Department of Pediatric Hematology and Oncology, University Children’s Hospital Tuebingen, Hoppe-Seyler-Str.1, 72076 Tuebingen, Germany; 2 Department of Internal Medicine VIII, University Hospital Tuebingen, Otfried-Mueller-Strasse 10, D-72076 Tuebingen, Germany; 3 German Cancer Consortium (DKTK), DKFZ partner site Tuebingen, Otfried-Mueller-Strasse 10, D-72076 Tuebingen, Germany; 4 Institute of Physiology, Department of Vegetative and Clinical Physiology, University of Tuebingen, Otfried-Mueller-Str. 27, 72076 Tuebingen, Germany

**Keywords:** Virotherapy, Measles vaccine virus, NK cells, Immunotherapy, Pediatric sarcoma

## Abstract

**Background:**

In pediatric sarcomas, outcomes of established therapies still remain poor, especially due to high-grade resistances to chemotherapeutic compounds. Taking novel biological approaches into account, virotherapy was found to be efficient in many pediatric sarcoma types. Also NK cell therapy was denoted to represent a promising upcoming strategy for pediatric sarcoma patients. We here investigated a combinatorial approach employing oncolytic measles vaccine virotherapeutics (MeV) together with activated human NK cells (or PBMCs).

**Methods:**

The human sarcoma cell lines A673 and HT1080 were used to evaluate the efficacy of this combinatorial treatment modality. Oncolysis was determined by measuring real-time cell proliferation using the xCELLigence RTCA SP system. Furthermore, expression of receptors on NK cells and the respective ligands on A673 cells was analyzed by flow cytometry. To measure the protein release of activated NK cells a LEGENDplex™ assay was performed.

**Results:**

Monotherapy with MeV led to a time- and dose-dependent oncolytic reduction of A673 and HT1080 sarcoma tumor cell masses. Concurrently, such MeV infections did not change the expression of NK cell ligands MICA/B, ULBP1, 2, and 3, CD112, and CD155. As shown by real-time proliferation assays, infections of A673 and HT1080 sarcoma cells with MeV followed by co-culture with activated NK cells or PBMCs led to enhanced sarcoma cell destruction when compared to the respective monotherapies. In parallel, this dual therapy resulted in an increased release of granzymes, perforin, and granulysin from NK cells. In contrast, expression of activation and ontogenesis receptors on NK cells was not found to be altered after co-culture with MeV-infected A673 sarcoma cells.

**Conclusions:**

Taken together, the combined treatment strategy comprising oncolytic MeV and activated NK cells resulted in enhanced oncolysis of A673 and HT1080 cells when compared to the respective monotherapies. In parallel, we observed an increased release of NK cell activation markers upon co-culture with MeV-infected A673 human sarcoma cells. These results support the onset of clinical trials combining oncolytic virotherapy with NK cell based immunotherapies.

## Background

Sarcomas account for about 10% of all newly diagnosed cancers in children and young adults under the age of 20. Although improvements in overall survival of pediatric sarcoma patients have been made due to advances in multi-agent chemotherapy regimens, little success has been seen especially in the treatment of metastatic and relapsed disease. Therefore, novel treatment approaches are urgently needed.

Oncolytic viruses (OV) are designed to selectively infect and kill cancer cells by intracellular replication and subsequent oncolysis while sparing normal tissues [1–4]. Based on promising results in preclinical studies, a multitude of different OV species are currently in early stage and advanced clinical development [5, 6]. The herpes simplex virus (HSV)-based virotherapeutic compound Imlygic™ already has been approved for patients suffering from advanced stage melanoma [7]. Moreover, a single shot high-dose application of a measles vaccine virus (MeV) encoding a marker protein (MeV-NIS) resulted in a long-term tumor remission for now more than five years in a patient suffering from advanced stage multiple myeloma [8].

In a previous preclinical study we could demonstrate that MeV also shows oncolytic activity in sarcomas of children [9]. Some sarcoma cell lines, however, displayed primary resistance to MeV-mediated oncolysis, indicating the need for novel combinatorial approaches.

Natural killer (NK) cells are emerging as a highly promising treatment strategy in sarcomas [10, 11]*.* Adoptive transfer of NK cells already has been tested in various clinical trials (e.g., NCT00582816, NCT01287104) and has emerged as a safe and potentially efficacious immunotherapy for cancer patients [12, 13].

The cytolytic activity of NK cells towards virus-infected or malignant cells is dependent on the balance between inhibitory and activating signals, which are provided when the activating receptors NKG2D, DNAM-1, and the natural cytotoxicity receptors (NCRs) NKp30, NKp44, and NKp46 bind their respective ligands. NKG2D reacts with the UL-16 binding proteins ULBP1–6 and stress-inducible MHC class I-related polypeptide sequences (MIC) A and B, which are expressed by tumor cells. Killing of target cells only occurs when activating signals outweigh inhibitory ones.

Ex vivo activated and expanded NK cells from peripheral blood demonstrated a powerful in vitro cytotoxicity against pediatric solid tumors, including Ewing sarcoma, rhabdomyosarcoma, and osteosarcoma [14–16]. Moreover, a substantial antitumor effect was achieved in a Ewing sarcoma xenograft mouse model, resulting in disease eradication in some animals [17]. NK cells constitute a dual function component of the innate immunity mediating not only potent tumor cell clearance but also antiviral immunity.

Viral replication and subsequent direct oncolysis lead to an increase in the expression of chemoattractants and activators of maturation for components of the innate immune system, including NK cells, macrophages, dendritic cells, and neutrophils, thus creating a pro-inflammatory environment [18]. Also, ongoing necrosis by viral oncolysis and the recruited components of innate immunity may facilitate an influx of de novo immune cells into the previously immune-protected tumor microenvironment.

Beyond that, it recently was found that NK cells became selectively cytotoxic towards tumor cells when activated by oncolytic reoviruses [19]. In contrast, it was shown in a mouse glioblastoma model that an oncolytic HSV virus leads to recruitment of activated NK cells which selectively lyse infected tumor cells thereby leading to rapid viral clearance and thus partially limiting the success of virotherapy [20]. Interestingly, when a similar oncolytic HSV virus was tested, now engineered to express E-cadherin (CDH1 gene), an adherent molecule and a ligand for KLRG1, an inhibitory receptor expressed on NK cells, a reduced viral clearance by selectively protecting OV-CDH1-infected cells from KLRG1^+^ NK cell killing was observed [21].

In the present study, we investigated a combinatorial approach of oncolytic MeV and activated NK cells in the treatment of human sarcoma cells. We found an enhanced rate of tumor cell destruction when compared to the respective monotherapies. In parallel, we observed an increased release of granzymes, perforin, and granulysin from NK cells upon co-culture with MeV-infected A673 human sarcoma cells.

## Methods

### Cell lines

Vero african green monkey kidney cells were obtained from the German Collection of Microorganisms and Cell Cultures (No. ACC 33; DSMZ, Braunschweig, Germany). Human A673 cells (extraosseous Ewing sarcoma; No. CRL-1598) and human HT1080 cells (fibrosarcoma; No. CCL-121) were purchased from the American Type Culture Collection (ATCC, Manassas, VA, USA). All cell lines were maintained in Dulbecco’s modified Eagle’s medium (DMEM, Sigma-Aldrich, Munich, Germany) supplemented with 10% fetal bovine serum (FBS, Sigma-Aldrich) at 37 °C in a humidified atmosphere containing 5% CO_2_ and mycoplasma testing was performed regularly every three months (MycoTOOL PCR Mycoplasma Detection Kit, Roche, Mannheim, Germany).

### Isolation of peripheral whole blood mononuclear cells (PBMCs)

PBMCs were isolated from healthy donors by density gradient centrifugation using Biocoll separating solution (Biochrom GmbH, Berlin, Germany) after informed consent. CD3^+^ cells were subsequently depleted by CD3 Dynabeads (Invitrogen, Carlsbad, CA, USA) according to the manufacturer’s protocol. CD3-depleted PBMCs were seeded at a cell density of 1.0 × 10^6^ in 25 ml culture flasks in RPMI 1640 medium (Biochrom) supplemented with 10% fetal bovine serum (Biochrom), 2 mM L-glutamine (Biochrom), 100 U/ml penicillin (Biochrom), and 100 μg/ml streptomycin (Biochrom) in the presence of 5% CO_2_ in a humidified atmosphere at 37 °C. All experiments involving human tissues were approved by the ethics committee at the Medical Faculty of the Eberhard Karls University and the University Hospital Tuebingen (349/2013BO) and informed consent was obtained from healthy donors in accordance with the Helsinki Declaration of 1975 (revised in 2008).

### Stimulation of PBMCs

CD3-depleted PBMCs were cultured in the absence or presence of human IL-2 in a concentration of 100 IU/ml for 24 h.

### Preparation of ex vivo activated and expanded NK cells (NKAES)

Freshly isolated PBMCs were co-cultured with 100 Gy irradiated K562mb15 4-1BBL feeder cells (kindly provided by Dario Campana). Cells were cultured in complete RPMI 1640 medium (Biochrom) containing 10% AB-human serum, 2 mM L-glutamine (Biochrom), 100 U/ml penicillin (Biochrom), 100 μg/ml streptomycin (Biochrom), and 100 IU/ml recombinant human IL-2 (Proleukine). Medium was changed every 2 to 3 days. The NKAES were harvested on days 10–15 and subsequently characterized by flow cytometry.

### Propagation and titration of measles vaccine virus

To prepare virus stocks, 5 × 10^6^ Vero cells were seeded in 15 cm plates (TPP, Trasadingen, Switzerland). The next day, cells were washed with phosphate buffered saline (PBS; Sigma-Aldrich) and infected for 3 h at a multiplicity of infection (MOI) of 0.03 in Opti-MEM® I (Invitrogen, Carlsbad, CA, USA). After infection, the inoculum was removed and DMEM supplemented with 10% FBS was added. At 54 h post infection (hpi), when most of the cells were infected, medium was removed, cells were scraped into 1 ml Opti-MEM® I, and the virus was released by one freeze/thaw cycle. After centrifugation (1900 x g, 15 min, 4 °C), the cleared supernatant was stored at − 80 °C. Viral titers were determined on Vero cells according to the method of Kärber and Spearman [22, 23].

### Virus infections

The day before virus infection, A673 and HT1080 cells were seeded in 6- or 24-well plates. Then medium was discarded and cells were washed once with PBS. MeV-GFP was diluted in Opti-MEM and added at the indicated MOIs. At 3 hpi the inoculum was removed and normal growth medium was added.

### Sulforhodamine B cell viability assay

Cells were seeded in 24-well plates (4 × 10^4^ cells/well) and infected with MeV-GFP on the following day at MOIs ranging from 0.1 to 10. At the indicated time points, cells were washed once with ice-cold PBS and fixed with 10% trichloroacetic acid (TCA) for 30 min at 4 °C. After washing with tap water and drying, proteins were stained for 10 min with Sulforhodamine B (SRB) staining solution (0.4% in 1% acetic acid) followed by washing with 1% acetic acid and drying again. Protein-bound dye was extracted with 10 mM Tris base (pH 10.5). After 10 min incubation at room temperature (RT) optical density was measured with a 96-well microtiter plate reader (Tecan Genios Plus, Tecan Deutschland, Crailsheim, Germany) at a wavelength of 550 nm (reference wavelength at 620 nm).

### Flow cytometry

Antibodies and their corresponding isotype controls were purchased from abcam (UK), BD Pharmingen (Germany), Beckman Coulter (Germany), BioLegend (Germany), eBioscience (USA), R&D (Germany), and Miltenyi Biotec (Germany). In any flow-cytometric analysis, live, vital cells were selected and doublets excluded based on scatter characteristics and low (auto-)fluorescence intensities after incubation with the Zombie-Aqua Fixable Viability Kit (BioLegend, USA). All samples were analyzed with the flow cytometer LSR II (Becton Dickinson, Germany) using BD FACSDiva software or with a FACS Attune NxT cytometer (Thermo Fisher Scientific, USA).

### Immunophenotyping

The following antibody clones were used for phenotypical NK cell characterization: CD3 (UCHT1), CD16 (3G8), CD25 (2A3), CD56 (HCD56), CD69 (L78), CD94 (HP-3D9), CD158a/b/e (HP-3E4), CD161 (HP-3G10), NKp30 (Z25), NKp44 (Z231), NKp46 (9E2/NKp46), NKG2A (Z199), NKG2C (134591), and NKG2D (BAT221). The percentage of CD56^+^CD3^−^-cells expressing each antigen was determined using cluster analysis. For detection of NK cell ligands A673 cells were seeded in 6-well plates (6 × 10^5^ cells/well) and infected with MeV-GFP at MOI 0.5. At 48 hpi cells were washed with PBS, detached using Accutase® (Sigma-Aldrich), and diluted in FACS buffer (PBS, 1% FBS). Cell surface molecules of tumor cells were characterized by flow cytometry using fluorochrome conjugated antibody clones CD112 (TX31), CD155 (SKII4), MICA/B (159207), PD-L1 (29E2A3), ULBP1 (170818), ULBP2/5/6 (165903) or ULBP3 (166510). Cells were stained for 30 min at 4 °C in the dark, washed with PBS, and fixed with 2% formaldehyde diluted in FACS buffer.

### Real-time cell monitoring assay

A673 cells (5 × 10^3^ cells/well) were seeded in 96-well plates (E-Plate 96, Roche Applied Science, Mannheim, Germany). Real-time dynamic cell proliferation was monitored in 30 min intervals during a 130 h observation period using the xCELLigence RTCA SP system (Roche Applied Science). Cell index values were calculated using the RTCA Software (1.0.0.0805). 21 h after seeding, cells were infected with MeV-GFP at MOI 0.5 or mock-infected. At 51 hpi PBMC, PBMC stimulated with IL-2 or NK cells were added at the indicated effector to target (E:T) ratios [24, 25]. HT1080 cells (1 × 10^3^ cells/well) were infected at 24 h after seeding with MeV-GFP at MOI 5 or mock-infected. At 23 hpi, NK cells were added at E:T ratios ranging from 1:1 to 5:1. Cell proliferation was monitored in 60 min intervals during a 96 h observation period.

### Analysis of culture supernatants

For analysis of culture supernatants A673 (6 × 10^5^ cells/well) cells were seeded in 6-well plates and infected with MeV-GFP at a MOI of 1. At 24 hpi NK cells were added at an E:T ratio of 2.5:1. 24 h later supernatants were collected and analyzed using the LEGENDplex™ kit (Human CD8/NK Panel) (BioLegend, San Diego, CA, USA) according to the manufacturer’s instructions.

### Statistical analysis

Statistical analysis was performed with GraphPad Prism Version 4.03 (GraphPad Software). A two-tailed unpaired *t* test was used to determine significance between two treatment groups. Reduction of cell mass was analyzed by one-way ANOVA and Dunnett’s multiple comparison test. Four different *p* values were determined: *p* < 0.05 (*), *p* < 0.01 (**), *p* < 0.001 (***), *p* < 0.0001 (****).

## Results

### Oncolytic activity of measles vaccine virus on sarcoma cells

To investigate a combinatorial approach employing oncolytic measles vaccine virus together with activated NK cells or PBMCs we used the human extraosseous Ewing sarcoma cell line A673 and the human fibrosarcoma cell line HT1080, which previously had been shown to be susceptible to MeV-mediated oncolysis when using our suicide gene-armed MeV (MeV-SCD) oncolytic virus. Susceptibility to virus-mediated oncolysis was defined by a remaining tumor cell mass below 50% at 96 h post infection (hpi) when using a multiplicity of infection (MOI) of 1 (i.e., application of one infectious viral particle per cultured tumor cell).

**Fig. 1:**
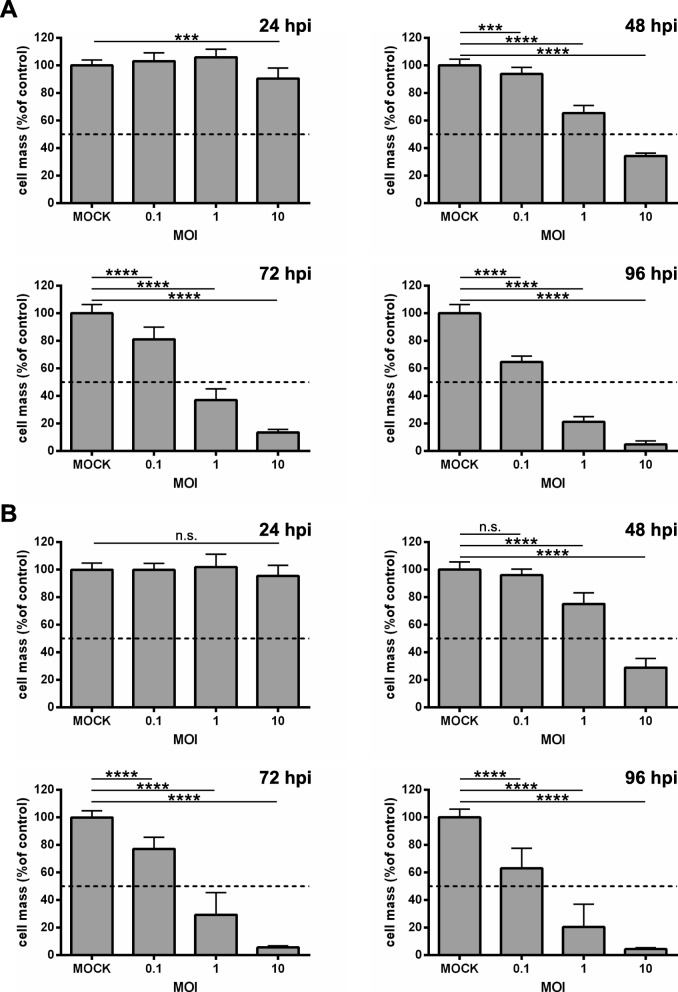
Viability of A673 (**a**) and HT1080 (**b**) sarcoma cell lines after infection with measles vaccine virus MeV-GFP. A673 (**a**) and HT1080 (**b**) cells were infected with MeV-GFP at multiplicities of infection (MOIs) of 0.1, 1, and 10, or MOCK-infected. At 24, 48, 72, and 96 h post infection (hpi) the remaining tumor cell mass was determined by SRB viability assay. MeV-GFP-mediated oncolysis is calculated relative to the MOCK-infected control. The mean ± SD of three independent experiments performed in quadruplicates is shown. * *p* < 0.05; ** *p* < 0.01, *** *p* < 0.001, **** *p* < 0.0001, n.s. not significant

To corroborate these results and to gain a more detailed insight into the course of infection we first infected both sarcoma cell lines with a GFP marker gene encoding measles vaccine vector (MeV-GFP) at MOIs 0.1, 1, and 10 and determined the remaining sarcoma cell masses at 24, 48, 72, and 96 hpi by a SRB viability assay (Fig. 1). As a result, in both cell lines a time- and MOI-dependent reduction of sarcoma cell masses could be observed when using MOIs of 1 and 10, starting at 48 hpi (Fig. 1 a, b). When employing MOIs 1 and 10 the remaining tumor cell masses were reduced to 37 and 13% at 72 hpi, respectively, in A673 cells (Fig. 1 a), and to 29 and 6% in HT1080 cells (Fig. 1 b). Notably, the lower MOI of 0.1 was found to reduce the tumor cell mass to 64% (A673 cells, Fig. 1 a) and 63% (HT1080 cells, Fig. 1 b) at 96 hpi, whereas MOIs 1 and 10 led to a further dramatic reduction of the remaining tumor cell masses in A673 cells at 96 hpi to 21 and 5% (Fig. 1 a). In HT1080 cell masses were reduced to 20% (MOI 1) and 4% (MOI 10), respectively (Fig. 1 b) at 96 hpi. Thus, our previous data, where A673 and HT1080 cells were classified as highly susceptible to MeV-mediated oncolysis, could be corroborated here in more detail.

### Expression of NK cell ligands on A673 cells

**Fig. 2:**
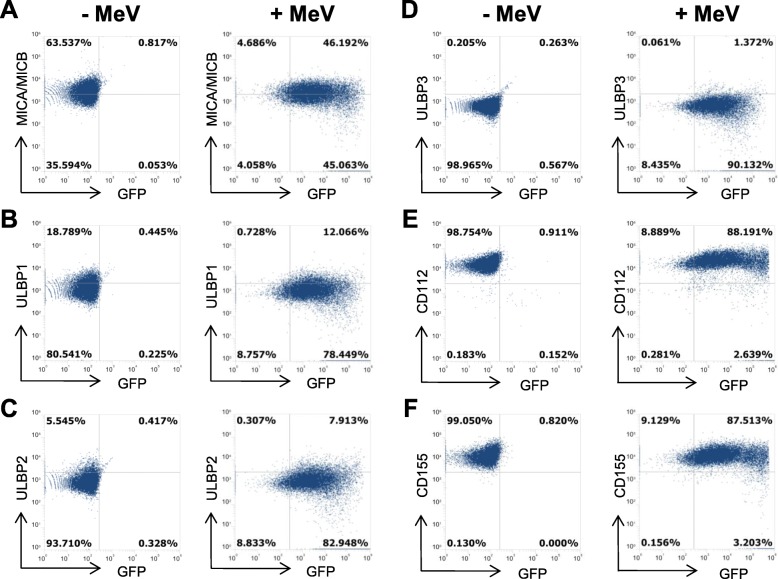
Expression of NK cell ligands on mock- vs. MeV-GFP-infected A673 sarcoma cells. A673 cells were mock-infected (left panels) or infected with MeV-GFP at MOI 0.5 (right panels). At 48 hpi expression levels of NK cell ligands MICA/MICB (**a**), ULBP1 (**b**), ULBP2 (**c**), ULBP3 (**d**), CD112 (**e**), and CD155 (**f**) were determined by flow cytometry. One representative of three independent experiments is shown. MeV, measles vaccine virus; GFP, green fluorescent protein

For cytotoxic activity of NK cells, the interaction of NK cell receptors with their respective ligands on target cells is indispensable. Therefore, we next investigated the influence of MeV infection on the expression of NK cell ligands on A673 sarcoma cells. For this purpose, sarcoma cells were infected with MeV-GFP at MOI 0.5 or mock-infected and then quantified for expression of NK cell ligands by flow cytometry. At 48 hpi, the remaining tumor cell mass was 70% at MOI 1 (Fig. 1 b) and expression of the marker protein GFP was close to 100% (Fig. 2; right panels) thus ensuring a sufficiently high amount of infected, but still viable tumor cells, being required for further combination experiments with NK cells.

Flow cytometry revealed expression of MICA and MICB on A673 cells which was not further enhanced by MeV infection. Whereas 64% of mock-infected A673 cells expressed MICA/MICB (Fig. 2 a, left panel; mock infection), 51% of A673 cells expressed MICA/MICB after MeV-GFP infection at 48 hpi (Fig. 2 a, right panel; infection with MeV-GFP). Expression of NKG2D ligands ULBP1, 2, and 3 was not significantly increased by MeV infection either (Fig. 2 b-d). In detail, percentage of ULBP1 positive A673 cells decreased from 19 to 13% (Fig. 2 b), for ULBP2 a minor increase from 6 to 8% could be observed at 48 hpi with MeV (Fig. 2 c). No expression of ULBP3 was detectable on mock-infected or MeV infected A673 cells (Fig. 2 d). CD112 and CD155 were constitutively expressed on A673 sarcoma cells at very high levels; accordingly, MeV infection did not change ligand expression (Fig. 2 e-f). Taken together, infection with MeV-GFP did not induce any significant alterations in the expression of NK cell ligands on A673 sarcoma cells.

### Induction of programmed death ligand 1 (PD-L1) expression on sarcoma cells upon MeV infection

**Fig. 3:**
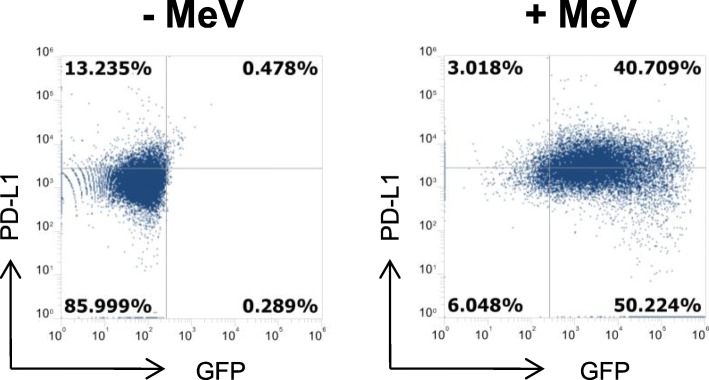
MeV-mediated induction of programmed death ligand 1 (PD-L1) expression on A673 cells. A673 cells were mock-infected (left panel) or infected with MeV-GFP (MOI 0.5) (right panel). At 48 hpi expression of PD-L1 was determined by flow cytometry. One representative of three independent experiments is shown

To examine the expression of the immune checkpoint ligand Programmed death ligand 1 (PD-L1) on mock-infected and MeV-infected A673 cells (Fig. 3) we used flow cytometry. Again, infection was performed with MeV-GFP at MOI 0.5; then, PD-L1 expression was analyzed at 48 hpi by flow cytometry. We found a strong induction of PD-L1 expression rising from a baseline of 14% (mock infection) to 44% (MeV infection) (Fig. 3) thus making human sarcoma cells prone for a combination therapy of MeV and immune-checkpoint inhibitors.

### Expression of activation and ontogenesis receptors on activated and expanded NK cells (NKAES) after co-culture with MeV-infected sarcoma cells

**Fig. 4:**
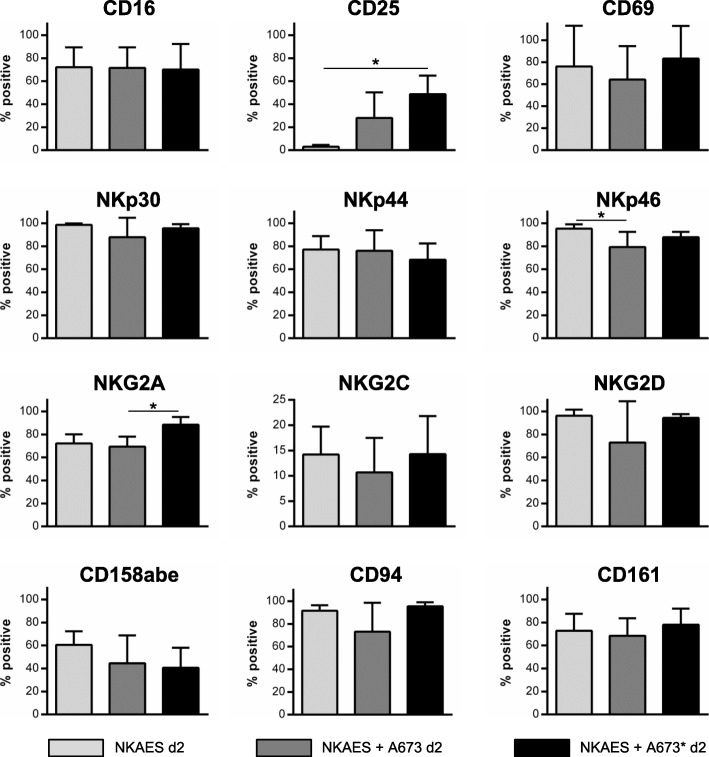
Characterization of NK cell receptors after co-culture of NKAES with MeV-infected A673 sarcoma cells. NKAES: activated and expanded NK cells; NKAES d2: on d2 without co-culture; NKAES+A673 d2: two days co-culture (E:T ratio = 2.5:1) with uninfected A673 sarcoma cells; NKAES+A673* d2: two days co-culture (E:T ratio = 2.5:1) with MeV-GFP infected A673 sarcoma cells (MOI 1). Samples were subjected to flow cytometric quantification of the proportion of cells expressing the given receptors. Bars represent the mean percentage of the respective CD56^+^ CD3^-^ NK cell subsets, error bars display SD. Note, that the CD56 receptor is not included in this diagram as all cells were gated on CD56 prior to subset analysis. Results represent data from 5 different donors. * *p* < 0.05

In a next step we studied the expression of activation and ontogenesis receptors on activated and expanded NK cells (NKAES) after co-culture with mock-infected or MeV-infected A673 cells (Fig. 4). For this purpose, A673 tumor cells were infected with MeV-GFP at MOI 1, which was chosen to obtain high amounts of infected cells already at 24 hpi. Then, at 24 hpi, NK cells were added to the mock-infected or MeV-infected sarcoma cells at an E:T ratio of 2.5:1. After 48 h of co-culture, percentages of cells expressing the respective receptor were determined by flow cytometry (Fig. 4). In parallel, receptor expression was analyzed on NKAES alone.

NKAES co-cultured with MeV-infected A673 sarcoma cells displayed phenotypical characteristics of rather advanced maturity which should go along with high levels of functional activities. Of note, maximum levels of receptor expression on NKAES could not be further elevated when co-cultured with MeV-GFP infected A673 sarcoma cells (except for NKG2A and CD25). Examination of surface markers revealed a CD56^dim^CD94^hi^CD16^hi^NKp46^hi^inhKIR^int^ fully mature NK cell phenotype on day 2 irrespective of the infection status of the co-cultured A673 sarcoma cells (Fig. 4). Therefore, NKAES co-cultured with MeV-infected A673 cells exhibited an inconspicuous phenotype resembling overall features of control NK cells or NK cells co-cultured with uninfected A673 cells.

### Co-culture with NK cells or PBMCs enhances oncolysis of MeV-infected sarcoma cells

We next set out to investigate whether combinatorial treatment with MeV and NK cells or PBMCs would result in higher oncolysis rates than MeV monotherapy in A673 and HT1080 sarcoma cells. This was done by measuring real-time cell proliferation using the xCELLigence RTCA SP system.

**Fig. 5:**
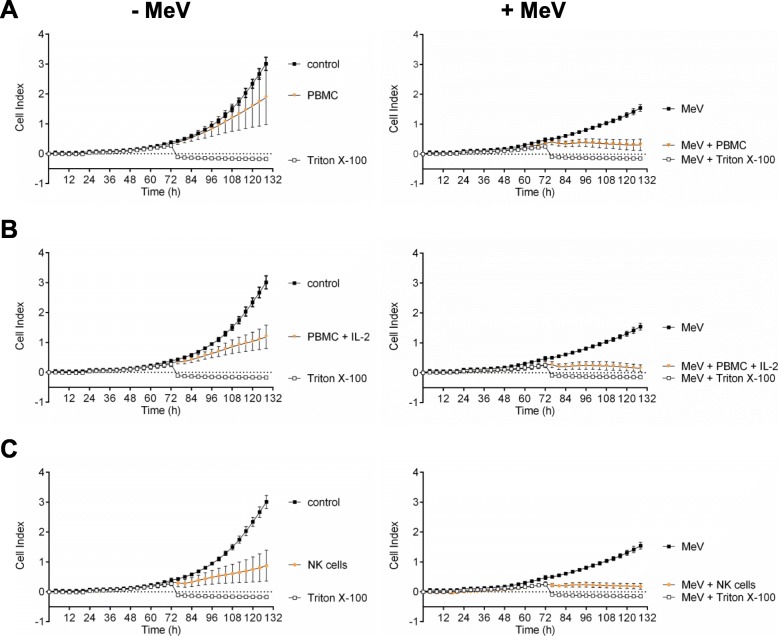
Real-time analysis of MeV-GFP-mediated oncolysis of A673 sarcoma cells after co-treatment with PBMC/NKAES isolated from a healthy donor. At 21 h after seeding, A673 cells were infected with MeV (MOI 0.5) (right panels) or mock-infected (left panels; base line controls). At 51 hpi, (**a**) PBMC, (**b**) PBMC stimulated with IL-2, or (**c**) NKAES from a healthy donor were added at an E:T ratio of 2.5:1. Triton X-100 was added as a negative control inducing maximum lysis of tumor cells. Real-time cell proliferation was monitored using the xCELLigence RTCA SP system. Measured electrode impedance is expressed as Cell Index. One representative of three independent experiments performed in triplicates using different donors is shown

First, A673 sarcoma cells were infected with MeV-GFP at 21 h after seeding (MOI of 0.5; Fig. 5, right panel) or mock-infected (Fig. 5, left panel). Then, at 51 hpi, (a) unstimulated PBMC, (b) PBMC stimulated with IL-2, or (c) NK cells all from the same healthy donor were added at an E:T ratio of 2.5:1. Finally, real-time cell proliferation was monitored until 107 hpi.

**Fig. 6:**
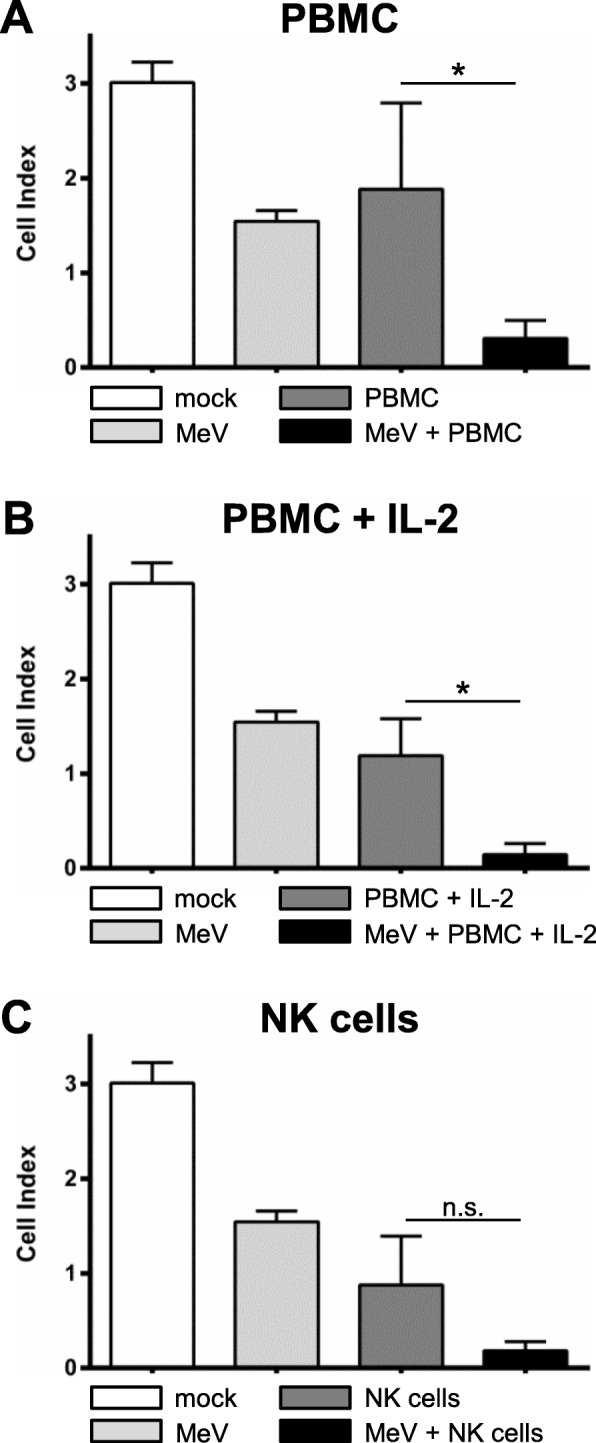
Statistical analysis of MeV-GFP-mediated oncolysis of A673 sarcoma cells after co-treatment with PBMC/NK cells isolated from a healthy donor. Analysis was performed as described in Fig. 5. Real-time cell proliferation is depicted as Cell Index after addition of PBMCs (**a**), PBMC stimulated with IL-2 (**b**), and NK cells (**c**) at 107 hpi. * p < 0.05; ** *p* < 0.01, *** *p* < 0.001, n.s. not significant

As a result, uninfected A673 sarcoma cells showed a profound unhindered tumor cell proliferation (Fig. 5, left panels; controls). Notably, addition of PBMC resulted only in a weak cytotoxic effect (Fig. 5 a, left panel). In contrast, MeV-infected A673 cells showed a weaker proliferation. Addition of PBMC to MeV-infected A673 cells led to a much higher cytotoxicity when compared to mock-infected cells (Fig. 5 a, right panel). Addition of PBMC stimulated with IL-2 (Fig. 5 b) or addition of NK cells (Fig. 5 c) resulted in an even higher cytotoxicity on the addressed sarcoma cells, whereby the cytotoxic effect again was significantly stronger on MeV-infected A673 cells (Fig. 5 b, c, right panel) than on mock-infected cells (Fig. 5b, c, left panel). The significances between each experimental group at 107 hpi were depicted in separate diagrams in Fig. 6.

Thus, combinatorial treatment of A673 sarcoma cells with oncolytic virus MeV-GFP and PBMCs stimulated with IL-2 or with NK cells was found to be highly superior when compared with the respective monotherapies.

**Fig. 7:**
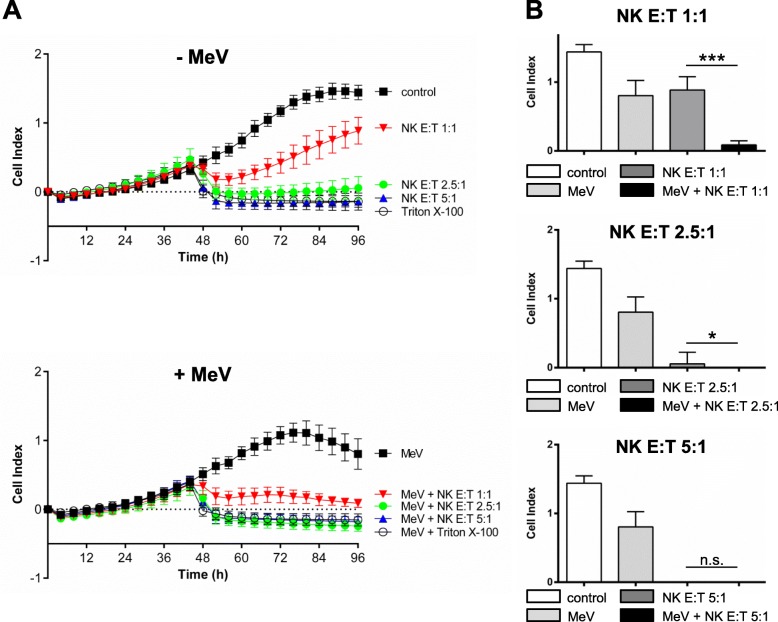
Real-time analysis of MeV-GFP-mediated oncolysis of HT1080 sarcoma cells after co-treatment with PBMC/NKAES isolated from a healthy donor. At 24 h after seeding, HT1080 cells were infected with MeV (MOI 5) (A, lower panel) or mock-infected (**a**, upper panel; base line controls). At 23 hpi, NK cells from a healthy donor were added at E:T ratios of 1:1, 2.5:1 and 5:1. Triton X-100 was added as a negative control inducing maximum lysis of tumor cells. Real-time cell proliferation was monitored using the xCELLigence RTCA SP system until 72 hpi. Measured electrode impedance is expressed as Cell Index. One representative of two independent experiments performed in quadruplicates using different donors is shown. (**b**) Statistical analysis of the same experiment. * p < 0.05; ** p < 0.01, *** p < 0.001, n.s. not significant

For HT1080 sarcoma cells a different regimen had to be chosen due to different growth characteristics of this cell line. HT1080 cells were infected at 24 h after seeding with MeV-GFP (MOI of 5, Fig. 7 a, lower panel) or mock infected (Fig. 7 a, upper panel). At 23 hpi, NK cells from a healthy donor were added at E:T ratios of 1:1, 2.5:1 and 5:1. Real time cell proliferation was monitored until 72 hpi. Infection of HT1080 with MeV-GFP resulted in a weak cytotoxic effect. Addition of NK cells at a low E:T ratio of 1:1 led to a significantly stronger cytotoxic effect on MeV-infected HT1080 cells when compared to uninfected cells. At an E:T ratio of 2.5:1 the effect of NK cells on MeV-infected HT1080 cells was still significantly stronger whereas at an E:T ratio of 5:1 both uninfected and MeV-infected HT1080 cells were completely lysed. The significances between experimental groups at 96 hpi were depicted in Fig. 7 b.

### Increased protein release from NK cells upon co-culture with MeV-infected sarcoma cells

**Fig. 8:**
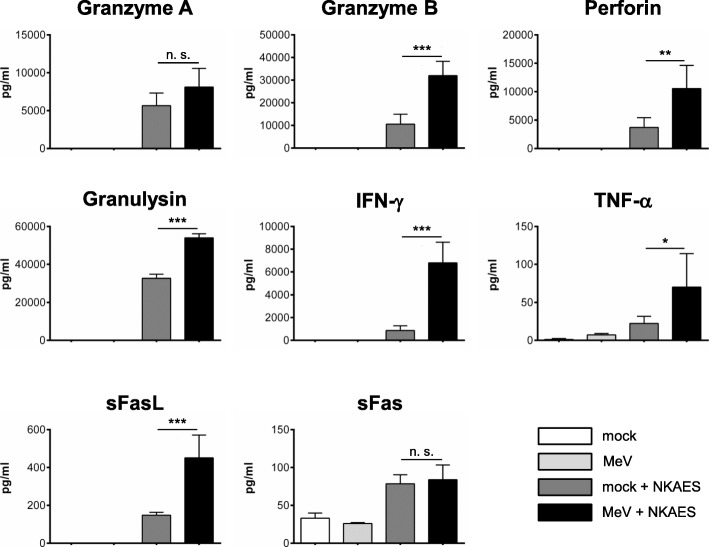
Quantification of protein release from NK cells after co-culture with MeV-infected A673 sarcoma cells. A673 sarcoma cells were infected with MeV-GFP (MOI 1) or mock-infected. At 24 hpi, NK cells from healthy donors were added at an E:T ratio of 2.5:1. Then, 24 h later, supernatants were collected and protein contents were determined by flow cytometry using LEGENDplex™ assay. A673 sarcoma cells without addition of NK cells were used as controls. The mean ± SD resulting from three different donors is shown. * *p* < 0.05; ** *p* < 0.01, *** *p* < 0.001, n.s. not significant

To gain a more detailed insight into the mechanism of enhanced killing of A673 sarcoma cells undergoing combination therapy (oncolytic virus MeV plus NK cells) we next had a look on the protein release from NK cells. For this purpose, A673 sarcoma cells were infected with MeV-GFP at MOI 1 or mock-infected. At 24 hpi, NK cells were added at an E:T ratio of 2.5:1. 24 h later supernatants were collected and analyzed by LEGENDplex™ assay (Fig. 8). Co-culture with MeV-infected A673 cells led to an increased release of granzymes A (8 ng/ml compared to 6 ng/ml) and B (32 ng/ml compared to 11 ng/ml), perforin (11 ng/ml compared to 4 ng/ml), and granulysin (54 ng/ml compared to 33 ng/ml) (Fig. 8, upper panel) being indicative of NK cell activation. None of these molecules was detectable in the supernatant of A673 cells cultivated in the absence of NK cells. Moreover, we also found higher amounts of IFN-γ (7000 pg/ml versus 865 pg/ml) and sFasL (450 pg/ml compared to 148 pg/ml) after co-culture of MeV-infected A673 cells with NK cells (than with mock-infected A673 cells only). In contrast, only minor amounts of TNF-α (< 100 pg/ml) and sFas (< 100 pg/ml) were detectable. As a result, increased cell killing of MeV-infected A673 sarcoma cells, cultivated together with NK cells, parallels increased release of NK cell activation markers.

Taken together, these data are very encouraging and support the onset of clinical trials combining MeV-based oncolytic virotherapy with NK cell-based immunotherapies.

## Discussion

Oncolytic immunovirotherapy is an emerging treatment modality for a variety of cancers. With regard to pediatric cancers, several OVs are currently under investigation [26] and already have shown promising antitumoral effects in numerous pediatric preclinical tumor models [27–30]. While clinical applications of virotherapeutics e.g. based on herpes simplex virus [31], reovirus [32], and vaccinia virus [33] have been demonstrated to be safe also in pediatric patients, disappointingly in none of the pediatric studies any objective responses could be obtained so far. In this situation, it is highly tempting to combine the profound immunostimulatory features of oncolytic virotherapy with the highly effective tumoricidal properties of immune cell-based therapies, such as NK cell-based therapies. Thereby, tumor cells potentially could be first “marked” immunologically by virotherapeutics, followed then by their highly efficient elimination by NK cells.

NK cells are, unlike T and B cells, components of the innate immune system and contribute to the first line of defense against cancer and viral infections. Their activation is determined by the balance of signals delivered by activating and inhibitory receptors, which is why NK cells can recognize a target without prior sensitization [34]. Activated NK cells execute their powerful cytotoxicity via multiple approaches, including direct lysis by granule-mediated cell apoptosis (release of perforin and granzymes), induction of apoptosis by FasL/Fas or tumor necrosis factor (TNF)-related apoptosis-inducing ligand (TRAIL)/TRAIL receptors, and the release of cytokines such as interferon (IFN)-γ and TNF-α to activate macrophages as well as antigen-specific cytotoxic T-cells [35–37]. Based on these functions, NK cells are regarded as powerful immune effectors in tumor surveillance and tumor control.

However, it also has been shown that NK cells often exhibit malfunctions in cancer patients and thereby can help tumors to escape immune surveillance [38]. Such immune escape scenarios potentially can be shattered and dissolved by virotherapy-induced tumor cell death (achieving a conjoint viral and tumoral antigen release in highly inflammatory milieus), which could help to restore the proper tumoricidal functionalities of NK cells. In detail, virotherapeutic-induced tumor cell death results in the release of virus-related pathogen-associated molecular patterns (PAMPs) or danger-associated molecular patterns (DAMPs) that are recognized by pattern recognition receptors, such as toll-like receptors (TLRs), located in the cytoplasm or on the cell surface [39]. Their engagement induces expression of inflammatory cytokines (e.g., IFN and TNF-α), which bind to receptors on other cells, resulting in recruitment and activation of innate immune cells, such as NK, NKT, and γδ T-cells [40, 41]. NK cells then are able to sense virotherapeutically infected cells either through direct interaction with PAMPs via TLRs or through recognition of viral and/or virus-induced ligands via activating NK cell receptors [42].

Another promising therapeutic strategy to counteract those immune escape scenarios is to restore NK cell antitumoral functions by supplementing ex vivo activated and expanded NK cells with the intent to reverse their malfunctions in cancer patients [38].

Currently, there are many clinical trials investigating the immunotherapeutic effect of NK cell restoration for the treatment of cancer; specifically, there are two ongoing clinical trials utilizing NK cells (NCT01807468, NCT02100891) including also pediatric solid tumors such as pediatric sarcomas.

In our preclinical study, we examined both strategies in cell culture to restore the potent tumoricidal functionalities of NK cells in the pediatric extraosseous Ewing sarcoma model A673 and in the human fibrosarcoma cell line HT1080 by oncolytic virotherapy with a novel measles vaccine virus expressing the GFP marker protein (MeV-GFP) [43] as monotherapy as well as in a combinatorial treatment modality of MeV-based virotherapy together with NK cell-based therapy. Notably, MeV-GFP exhibits an outstanding safety profile, especially due to the fact that its backbone is 100% identical to the well-known measles vaccine virus *Schwarz* strain being in use for many decades for worldwide vaccination purposes. Accordingly, future applications of MeV-GFP and derived virotherapeutic vectors in pediatric tumor patients should meet the specific regulatory requirements placed on the treatment of tumor-bearing children.

When challenging A673 and HT1080 cells with MeV-GFP we could show that both sarcoma cell lines are highly susceptible to MeV-mediated oncolysis. This virotherapeutically achieved tumor cell mass reduction was demonstrated to be dependent on the amount of infectious virus particles being applied (i.e., the MOI used) as well as on the time point of infection and the duration of the respective infections.

The interaction of NK cell receptors with their respective ligands on target cells is a prerequisite for their cytotoxic activity. Recently, it was shown that infections of hepatocellular carcinoma (HCC) cells with the oncolytic measles vaccine virus strain *Edmonston* upregulated the expression of NK cell ligands MICA/B and therefore enhanced the cytotoxic activity of CD8^+^NKG2D^+^ cells in these HCC cell lines [44]. In our study, FACS analysis demonstrated that tumor cell infection with MeV-GFP did not induce any significant alterations or desired upregulation of the NK cell ligands investigated here on A673 sarcoma cells. However, the ligands MICA and MICB, as well as CD112 and CD155 were found to be constitutively expressed at high levels, indicating optimal conditions for efficient NK cell-based therapy already upfront of any therapeutic interventions.

In a next experimental setting, the combinatorial treatment modality of MeV-GFP virotherapy and NK cell based therapy with NKAES was examined under real-time conditions. Remarkably, xCELLigence data revealed that this combination therapy resulted in significantly higher rates of oncolysis in MeV-infected A673 and HT1080 sarcoma cells than any of the respective monotherapies (i.e., treatment with MeV-GFP alone or with NK cells alone).

In order to further examine the details for the observed increased antitumoral efficacy, the expression of activation and ontogenesis receptors on NKAES being cultivated in co-culture with A673 sarcoma cells was analyzed. Remarkably, a fully mature NK cell phenotype (CD56^dim^CD94^hi^CD16^hi^NKp46^hi^inhKIR^int^) was displayed which was not altered when MeV-infected A673 cells were used in this functional assay. This result indicates that the increased cytotoxic activity observed against A673 cells could not be explained by virus-induced upregulation of the activation and ontogenesis receptors on NK cells analyzed in this study. Notably, in a different preclinical study employing an oncolytic vesicular stomatitis virus (VSV) it was shown that interleukin-28 (Type III IFN) mediates the antitumoral efficacy of VSV by sensitizing tumor cells to NK cell recognition and activation [45]. Furthermore, preclinical research on an oncolytic reovirus revealed that NK cells became selectively cytotoxic towards tumor cells when being activated by reovirus. Interestingly, increased cytolytic activities of NK cells were found to be mediated by direct activation of human dendritic cells (DC) and upregulation of IFN-γ production [19, 46]. In this light, the exact mechanisms underlying the combinatorial effect of MeV-GFP and NK cell therapy (NKAES) in our experiments on A673 sarcoma cells should be elucidated in future studies.

To further investigate the involvement of NK cells in enhanced oncolysis, protein release from human NK cells after co-culture with MeV-infected A673 cells was determined and quantified. Interestingly, high amounts of granzymes A and B, perforin, and granulysin were released from virus-activated NK cells indicating their potent cytotoxic activity via granule-mediated cell apoptosis. Moreover, increased amounts of sFasL, also indicative for apoptosis, and IFN-γ, a cytokine which is known to play an important role in the induction of an adaptive immune response, were detected after co-culture of NK cells with MeV-infected A673 cells. These data demonstrate that NK anti-tumor reactivity appears to be modulated at the level of recognition although the identification of NK cell receptors/ligands which are specifically modulated by MeV infection and finally mediate this phenomenon is part of ongoing studies. Thereby, increased killing of MeV-infected A673 sarcoma cells, which were cultivated together with NK cells, parallels increased release of NK cell activation markers.

In the research field of virotherapy it becomes increasingly clear that any monotherapies with oncolytic viruses are not efficient enough in the treatment of cancer. Therefore, combination strategies with other cancer therapeutics are in the focus of clinical research, which so far revealed immune-checkpoint inhibitors (ICIs) as the most promising combination partners [47]. Interestingly, we found a strong induction of PD-L1 expression on MeV-GFP-infected A673 sarcoma cells which builds up a rationale for a multimodal therapeutic approach combining virotherapy and NK cell-based therapy together with immune-checkpoint inhibition in the future.

This idea of triple combination therapies with already approved cancer therapeutics is not new and so far already has shown promising results in preclinical studies. A much-discussed approach is the combination of HSV-based virotherapy together with the proteasome inhibitor bortezomib and NK cell immunotherapy. In an in vivo glioblastoma model, combinatorial treatment of bortezomib and HSV virotherapy significantly enhanced NK cell activation and adjuvant NK cell therapy further improved antitumoral efficacy. The authors explain this effect by increased surface expression levels of NK cell-activating markers and enhanced proinflammatory cytokine secretion induced by combined treatment of cancer cells with bortezomib followed by HSV [48]. Based on these results a follow up study investigated the complex role of NK cells in the regulation of the virotherapy/bortezomib combination therapy [49]. Kim and colleagues discovered that the antitumoral efficacy increases when on the one hand endogenous NK cells are depleted and on the other hand externally activated NK cells are injected directly into tumors. They postulate that patient’s own NK cells, which are present in smaller numbers, mainly concentrate on clearing virus infection and consequently have an adverse effect on virotherapy. This effect can be reversed by substituting higher numbers of externally activated NK cells which display rapid and potent antitumoral functions to overcome immunosuppressive tumor microenvironments [49].

At this point it should be mentioned that in this preclinical study the interaction of MeV-based virotherapy together with NK cell-based therapy exclusively was investigated in cell culture. Both therapeutic strategies are immunotherapies that require a functioning immune system to be fully effective. Therefore, it is of great importance to verify the observed cell-based effects also in immuno-competent animal models of pediatric sarcomas. With regard to the development of new therapeutics, potential barriers between cell-based and animal-based studies have to be identified and overcome.

## Conclusions

Interactions of NK cells with various elements of the tumor microenvironment as well as their possible effects in contributing to and/or limiting oncolytic virotherapy seem to be complex in nature; therefore, it is of great importance to dig deeper into the exact mechanisms of such interactions. In this context, we found an enhanced rate of tumor cell destruction using a combinatorial approach of oncolytic MeV and activated NK cells in the treatment of human sarcoma cells when compared to the respective monotherapies. Furthermore, we observed an increased release of granzymes, perforin, and granulysin from NK cells upon co-culture with MeV-infected A673 human sarcoma cells. These data demonstrate that a synergistic approach involving oncolytic virotherapy and NK cell-based immunotherapy provides a promising combined cancer therapy strategy and could pave the way for triple combinational approaches including e.g. immune-checkpoint inhibitors in the future.

## Data Availability

All datasets generated or analyzed during this study are available from the corresponding author on reasonable request.

## Abbreviations

CD: Cluster of differentiation
DAMP: Danger-associated molecular pattern
DMEM: Dulbecco’s modified eagle’s medium
DNAM-1: DNAX accessory molecule-1
FACS: Fluorescence-activated cell sorting
FBS: Fetal bovine serum
hpi: Hours post infection
HSV: Herpes simplex virus
IFN: Interferon
IL-2: Interleukin-2
KLRG1: Killer cell lectin-like receptor subfamily G member 1
MDSC: Myeloid-derived suppressor cell
MeV: Measles vaccine virus
MeV-GFP: Measles vaccine virus encoding green fluorescent protein
MeV-NIS: Measles vaccine virus encoding sodium-iodide symporter
MICA/B: MHC class I chain-related protein A/B
MOI: Multiplicity of infection
NK cells: Natural killer cells
NKAES: Activated and expanded NK cells
OV: Oncolytic virus
PAMP: Pathogen-associated molecular pattern
PBMCs: Peripheral blood mononuclear cells
PBS: Phosphate buffered saline
PD-L1: Programmed death ligand 1
RPMI: Cell culture medium developed at the Roswell Park Memorial Institute
RT: Room temperature
SRB: Sulforhodamine B
TCA: Trichloroacetic acid
TNF: Tumor necrosis factor
TRAIL: TNF-related apoptosis-inducing ligand
ULBP1/2/3: UL16-binding protein 1/2/3
